# Antileishmanial and cytotoxic activities of four Andean plant extracts from Colombia

**DOI:** 10.14202/vetworld.2020.2178-2182

**Published:** 2020-10-19

**Authors:** Wilson Cardona-G, Sara Robledo, Fernando Alzate, Andrés F. Yepes, Cristian Hernandez, Ivan Dario Velez, Juan Carlos Calderon, Isabel Vásquez Tabares

**Affiliations:** 1 Chemistry of Colombian Plants, Institute of Chemistry, Natural and Exact Sciences Faculty, Universidad de Antioquia, Colombia; 2 Group of Botanical Studies, Institute of Biology, Natural and Exact Sciences Faculty, Universidad de Antioquia, Colombia; 3 PECET, Medical Research Institute, School of Medicine, Universidad de Antioquia, Colombia

**Keywords:** Araliaceae, Chrysobalanaceae, Lauraceae, Rubiaceae, Leishmaniasis, *Licania salicifolia*, *Oreopanax floribundus*, *Persea ferruginea*, *Psychotria buchtienii*

## Abstract

**Background and Aim:**

*Licania salicifolia* (L.S) Cuatrec., *Persea ferruginea* (P.F) Kunth, *Oreopanax* *floribundus* (O.F), and *Psychotria buchtienii* (P.B) belong to the families Chrysobalanaceae, Lauraceae, Araliaceae, and Rubiaceae, respectively, which have been used as medicines by communities in the Andes. This study evaluated the leishmanicidal and cytotoxic activities of alcohol and non-alcohol extracts from four Andean plant extracts (L.S, O.F, P.F, and P.B).

**Materials and Methods:**

Extracts were obtained by percolation with solvents of different polarities — hexane, dichloromethane, ethyl acetate, and ethanol. Phytochemical screening was conducted based on reported methods. All products were evaluated *in vitro* to determine the leishmanicidal activity against amastigotes of *Leishmania panamensis* and cytotoxicity against U937 cells.

**Results:**

Flavonoids, triterpenes, and tannins were the main secondary metabolites found. From the results, dichloromethane extracts from O.F and P.B, ethanol extract from P.B, and ethyl acetate extracts of all plants were active, with EC_50_ <30 μg/mL. Ethyl acetate was the most active extract, which showed EC_50_ values of 9.8, 14.1, 23.7, and 25.5 μg/mL, for L.S, P.B, O.F, and P.F, respectively. Hexane extracts from P.B and O.F exhibited moderate activity with EC_50_ values of 84.8 and 87.4 μg/mL, respectively. Hexane and ethanol extracts from O.F, ethyl acetate, and ethanol extracts from L.S, and all extracts from P.F were not toxic. Alternatively, hexane and dichloromethane extracts from L.S and P.B as well as dichloromethane and ethyl acetate extracts from O.F displayed high toxicity.

**Conclusion:**

Based on the activity we observed, ethyl acetate extract can continue in its usage in the search for new antileishmanial drugs, mainly ethyl acetate extract from L.S showed activity comparable to meglumine antimoniate and was not cytotoxic.

## Introduction

Leishmaniasis is a group of diseases caused by protozoan parasites of the genus *Leishmania*, which infect and replicate inside macrophages of the vertebrate host. These diseases are considered a major health problem because of its presence in 98 countries, affecting mostly low-income people in rural areas of tropical and subtropical countries [1].

*Leishmania* (*Viannia*) *panamensi*s is an important causal agent of cutaneous leishmaniasis (CL) in Central and South America [2] and approximately 0.7-1.2 million cases occur annually. Different forms of leishmaniasis demand expensive treatments, the currently used medicines pentavalent antimonials, pentamidine isethionate, and miltefosine show high toxicity; therefore, they induce severe side effects. Thus, there is an urgency to develop new drugs to treat CL [3].

In many tropical countries, the treatment of leishmaniasis usually involves the oral administration of crude plant extracts for the systemic form of the disease and topical preparations of the corresponding extracts for treating skin infections [4]. Natural products, especially those derived from plants, are considered an important source of biologically active compounds against various infectious organisms, especially parasites. Many studies related to the activity of plant extracts against leishmaniasis have been reported [5].

Communities in the Andes have taken advantage of the availability of medicinal plants and have especially used aromatic plants such as those belonging to the families Chrysobalanaceae, Lauraceae, Araliaceae, and Rubiaceae [6]. Species such as *Licania salicifolia* (L.S) Cuatrec., *Persea ferruginea* (P.F) Kunth, *Oreopanax floribundus* (O.F), and *Psychotria buchtienii* (P.B), which belong to the aforementioned families, are part of these phytotherapeutic resources [7]. These plants exist in Andean forests at middle and high elevations between 1000 and 3000 m [8]. The antiprotozoal activity of these plant families has been published [9-13].

Based on these reports, we studied extracts of these plants to validate its traditional uses and to develop new chemotherapeutic agents against this protozoal disease.

## Materials and Methods

### Ethical approval

According to Colombian legislation, since this work involves only *in vitro* studies that do not require the use of animals or humans, the endorsement by the ethics committee is not required. 

### Study period and location

This study was carried out from June 2014 to December 2015 at the University of Antioquia, Medellin, Colombia.

### Plant materials

Plant material of L.S Cuatrec., O.F (Kunth) Decne. and Planch. (O.F), P.F Kunth and P.B (H.J.P. Winkl.) Standl (P.B) was collected during August and September 2013, in the eastern of the Department of Antioquia, Colombia (Table-1). Framework contract No. 234, RGE 289. Voucher specimens were kept at the University of Antioquia Herbarium (HUA)

**Table 1:** Plant material collection site.

Plant material	Department	Municipality (Temp)	Inclusion number	Latitude	Longitude
*Oreopanax floribundus* (Kunth) Decne. and Planch.	Antioquia	Medellín (20°C)	158049	6.182855555555556	-75.68648611111111
*Psychotria buchtienii* (H.J.P. Winkl.) Standl.	Antioquia	San Rafael (25°C)	177048	6.350833333333333	-74.99222222222222
*Licania salicifolia* Cuatrec	Antioquia	Rionegro (20°C)	156199	6.166666666666667	-74.36666666666666
*Persea ferruginea* Kunth	Antioquia	Belmira (20°C)	195102	6.6419444444444435	-75.71638888888889

### Extraction

The plant materials were dried in an oven at 35°C for 48 h. Powdered leaves of L.S (300 g), P.F (530 g), O.F (685 g), and P.B (430 g) were successively extracted with hexane, then dichloromethane, ethyl acetate, and finally ethanol in a percolator, at room temperature after which they were concentrated in a vacuum to give the corresponding extracts.

### Phytochemical screening

The phytochemical composition of the different plant extracts used in this study was analyzed. To detect steroids, triterpenoids, phenolics, flavonoids, alkaloids, saponins, anthraquinones, coumarins, and anthocyanosids, the method described by Londoño *et al*. was used [14].

### Biological activity assays

Extracts were subjected to *in vitro* evaluation to determine their cytotoxicity on U937 human cells and antileishmanial activity on intracellular amastigotes of *L*. (*V*) *panamensis*.

### *In vitro* cytotoxicity

The cytotoxic activity of extracts was assessed based on the viability of the human promonocytic cell line U937 (ATCC CRL-1593.2) evaluated using the (3-(4,5-dimethylthiazol-2-yl)-2,5-diphenyltetrazolium bromide) (MTT) assay following the methodology described previously [15]. Briefly, cells were grown in 96-well cell-culture dishes at a concentration of 100,000 cells/mL in Roswell Park Memorial Institute (RPMI) 1640 medium supplemented with 10% fetal bovine serum and the corresponding concentrations of the extracts, starting at 200 μg/mL in duplicate. Cells were incubated at 37°C and 5% CO_2_ for 72 h in the presence of the extracts and then the effect of the extracts was determined by adding 10 μL/well of MTT solution (0.5 mg/mL) and incubating at 37°C for 3 h. The reaction was stopped by adding 100 μL/well of 50% isopropanol solution with 10% sodium dodecyl sulfate and incubating for 30 min. Cell viability was determined based on the quantity of formazan produced according to the color intensity (absorbance) — registered as optical density (O.D) — obtained at 570 nm in a Varioskan LUX multimode microplate reader (Thermo Fisher Scientific, San Francisco, CA, USA). Cultured cells in the absence of the extracts were used as control of viability (negative control), while doxorubicin was used as a positive control for toxicity. The cytotoxicity of two conventional drugs (meglumine antimoniate and Amphotericin B) was also determined. Assays were conducted in duplicates with three replicates per concentration tested.

### *In vitro* antileishmanial activity

The activity of the extracts was evaluated using intracellular amastigotes of *L*. (*V*) *panamensis* transfected with the green fluorescent protein gene (MHOM/CO/87/UA140pIR-GFP) [15]. The effect of each extract was determined based on infection inhibition, which is evidenced by a decrease in the number of live intracellular amastigotes. Briefly, U937 human cells at a concentration of 3×10^5^ cells/mL in RPMI 1640 and 0.1 μg/mL of phorbol-12-myristate-13-acetate were infected with promastigotes in the stationary growth phase using 15:1 parasites per cell ratio and incubated at 34°C and 5% CO_2_ for 3 h. Cells were washed twice with phosphate-buffered saline (PBS) to eliminate non-internalized parasites. Fresh RPMI 1640 (1 mL) was added and cells were incubated again to guarantee the multiplication of intracellular parasites.

After 24 h of infection, the culture medium was replaced by a fresh culture medium containing each extract at four concentrations (100, 25, 6.25, and 1.78 μg/mL) or lower, (based on the cytotoxicity showed previously by each extract). After 72 h, the progress of infection inhibition was determined. Cells were removed from the bottom plate with a trypsin/ethylenediaminetetraacetic acid (250 mg) solution. Recovered cells were centrifuged at 1100 rpm at 4°C for 10 min, the supernatant was discarded and cells were washed with 1 mL of cold PBS and centrifuged at 1100 rpm at 4°C for 10 min. The supernatant was discarded and cells were suspended in 500-μL PBS and analyzed by flow cytometry (FC 500MPL, Cytomics, Brea, CA, US) with a total count of 10,000 events. The activity of tested extracts was conducted synchronously with the infection progress in culture medium alone as well as in culture medium with Amphotericin B and meglumine antimoniate as leishmanicidal drugs (positive controls). Determinations for each extract and standard drugs were conducted in triplicate in two independent experiments [15].

### Statistical analysis

Cytotoxicity was determined according to cell mortality percentages obtained for each isolated experiment (extracts, AmB, Sbv, and culture medium alone). Results were expressed as median lethal concentrations (LC_50_), and was calculated by Probit analysis [16] using the percentage of mortality, calculated using the formula in Equation 1, where the O.D of the control corresponds to 100% of viability (Cell growth).

% Mortality = 1 – [(O.D Exposed cells)/ (O.D Control cells)×100] (1)

The antileishmanial activity was determined according to the percentage of infected cells and parasite load obtained for each experimental condition using flow cytometry. The results of antileishmanial activity were expressed as the median effective concentration (EC_50_) determined by the probit method [16] using the percentage of viable intracellular parasites, calculated using the formula in Equation 2, where the mean fluorescence intensity (MFI) of the control-infected cells corresponds to 100% of parasitemia.

% Parasitemia = 1 – [(MFI Exposed parasites) /(MFI Control parasites) × 100] (2)

The cytotoxicity was graded according to the LC_50_ value (high cytotoxicity: LC_50_ <100 μg/mL, moderate cytotoxicity: LC_50_ >100-<200 μg/mL, and potentially no cytotoxicity: LC_50_ >200 μg/mL). In turn, leishmanicidal activity was graded according to the EC_50_ or IC_50_ value (high activity: EC_50_ <20 μg/mL, moderate activity: EC_50_ >20-<50 μg/mL, and potentially non-activity: EC_50_ >100 μg/mL).

The selectivity index (SI) was calculated by dividing the cytotoxic activity and the leishmanicidal activity using the following formula:

SI = LC_50_/EC_50_

Experiments were repeated at least 3 times for each concentration. Statgraphics Plus program version 5.0 (Statistical Graphics Corp., Rockville, MD) was used for all regression calculations with a significance level of p<0.05.

## Results

### Extraction and phytochemical screening

Extraction yield and phytochemical results for hexane, dichloromethane, ethyl acetate, and ethanol extracts for L.S*,* P.F*,* O.F, and P.B are reported in Table-2.

**Table 2:** Extraction yields and phytochemical constituents of the plants under study.

Plant extract	Yield (%)	Phytochemical profile
*Licania salicifolia*		
Hexane	(7.7 g) 2.6	Triterpenes
Dichloromethane	(5.3 g) 1.8	Triterpenes
Ethyl acetate	(2.3 g) 0.8	Triterpenes, flavonoids
Ethanol	(51.1 g) 17.0	Phenols, saponins, flavonoids, triterpenes, tannins, leucoanthocyanidins, anthocyanidins, coumarins
*Persea ferruginea*		
Hexane	(7.4 g) 1.4	Triterpenes
Dichloromethane	(3.7 g) 0.7	Triterpenes, coumarins
Ethyl acetate	(6.4 g) 1.2	Triterpenes, leucoanthocyanidins, coumarins
Ethanol	(45.1 g) 8.5	Alkaloids, flavonoids, saponins, tannins, leucoanthocyanidins, coumarins
*Oreopanax floribundus*		
Hexane	(10.3 g) 1.5	Triterpenes, flavonoids
Dichloromethane	(40.7 g) 5.9	Triterpenes
Ethyl acetate	(5.7 g) 1.0	Triterpenes, flavonoids
Ethanol	(42.5 g) 6.2	Saponins, flavonoids, tannins, leucoanthocyanidins
*Psychotria buchtienii*		
Hexane	(65.0 g) 15.1	Triterpenes, flavonoids
Dichloromethane	(11.08 g) 2.6	Triterpenes, flavonoids
Ethyl acetate	(2.3 g) 0.5	Triterpenes, saponins, anthocyanins, coumarins
Ethanol	(18.6 g) 4.3	Saponins, phenols, tannins, anthocyanins, coumarins

### Leishmanicidal activity and cytotoxicity

The effects of extracts on cell growth (viability) were assessed in human macrophages (U937 cells), which are the host cells for *L*. (*V*) *panamensis* parasites. Alternatively, the antiparasitic activity of these extracts was tested on intracellular amastigotes of *L*. (*V*) *panamensis* according to their ability to reduce the number of parasites after exposure. The results are summarized in Table-3.

**Table 3:** *In vitro* antileishmanial activity and cytotoxicity of the plant extracts under study.

Extract/control	Cytotoxicity, LC_50_ (μg/mL)^a^				Leishmanicidal activity, EC_50_ (μg/mL)^b\^				SI (LC_50_/EC_50_)			
		
	L.S.	P.F.	O.F.	P.B.	L.S.	P.F.	O.F.	P.B.	L.S.	P.F.	O.F.	P.B.
Hexane	54.0±4.4	>200	>200	11.6±1.50	>20	>20	87.4±7.6	84.8±2.50	<2.7	<10	>2.3	0.14
Dichloromethane	57.4±7.3	>200	47.4±4.6	76.8±2.80	>20	48.0±7.3	24.6±1.1	21.5±2.79	<2.9	>4.2	2.0	3.57
Ethyl acetate	>200	>200	51.1±5.9	109.5±7.50	9.8±1.2	25.5±2.6	23.7±1.0	14.1±0.47	>20.4	>7.8	2.2	7.75
Ethanol	>200	>200	>200	>200	>20	>20	>20	29.4±0.50	<10	<10	<10	>6.81
Sb(V)c	495.9±55.6				6.3±0.9				78.6			
Amphotericin B	42.1±2.0				0.04±0.01				1052.5			

L.S=*Licania salicifolia,* P.F=*Persea ferruginea,* O.F=*Oreopanax floribundus,* P.B*=Psychotria buchtienii*. Data represent the mean value±Standard deviation, ^a^LC_50_=Lethal concentration 50, Cytotoxic extract: LC_50_ <100 μg/ml. No cytotoxic extract=LC_50_ >200 μg/ml. ^b^EC_50_: Effective concentration 50, Leishmanicidal active extract: EC_50_ <50 μg/ml. Moderately active extract: EC_50_ <100 μg/ml, No Active extract: EC_50_ >100 μg/ml; ^c^SbV: pentavalent antimonial meglumine antimoniate

## Discussion

Table-2 shows that the best results were recorded for ethanol extracts from L.S*,* P.F, and O.F with a percentage yield of 17.0, 6.2, and 8.5%, respectively, and hexane extract from P.B with a percentage yield of 15.1%. The preliminary phytochemical analysis of the extracts revealed triterpenes in hexane, dichloromethane, and ethyl acetate extracts, while, tannins, leucoanthocyanidins, and saponins were only present in ethanol extracts of all plants. Alkaloids were found only in the ethanol extract from P.F. The presence of coumarins was found in the ethanol extract from L.S, as well as in ethyl acetate and ethanol extracts from P.F and P.B.

According to the results shown in Table-2, dichloromethane extracts from O.F and P.B, ethanol extract from P.B, and ethyl acetate extracts of all plants were active with EC_50_ <30 μg/mL. Ethyl acetate was the most active extract, which showed EC_50_ values of 9.8, 14.1, 23.7, and 25.5 μg/mL, for L.S, P.B, O.F, and P.F, respectively. Hexane extract from P.B and O.F exhibited moderate activity with EC_50_ values of 84.8 and 87.4 μg/mL, respectively. The activity of these extracts was probably due to the presence of compounds such as terpenoids, flavonoids, and coumarins (Table-1), which have long been recognized for their antiprotozoal activity [17-20].

Unfortunately, hexane extracts from L.S and P.F, dichloromethane extract from L.S, and ethanol extract from L.S, P.F, and O.F displayed leishmanicidal activity but at concentrations toxic to U937 cells, which serve as host cells of *Leishmania* parasites (EC_50_ >20 μg/mL).

Hexane and ethanol extracts from O.F, ethyl acetate and ethanol extracts from L.S, ethanol extract from P.B, all extracts from P.F, and pentavalent antimonial (meglumine antimoniate) were not toxic. Alternatively, hexane, and dichloromethane extracts from *L. salicifolia* and P.B as well as dichloromethane and ethyl acetate extracts from O.F displayed high cytotoxicity in a manner similar to Amphotericin B.

Except for the hexane extract from P.B, all SIs suggest that these extracts are selective since they were more active against *L*. (*V*) *panamensis* than U937 cells. Leishmanicidal activity displayed by these extracts against intracellular amastigotes of *L*. (*V*) *panamensis* suggests that they are promising candidates in the search for new antileishmanial compounds, mainly ethyl acetate extract from L.S, exhibited high SI and activity comparable to the conventional drug - meglumine antimoniate.

## Conclusion

The antileishmanial and cytotoxic screening of 16 extracts from four species of Colombian plants were reported. Based on activities observed, ethyl acetate extracts from all plants can continue in its usage in the search for new antileishmanial drugs, although further studies are required.

Different lineages of angiosperms produce useful metabolites for treating tropical diseases. In this study, it was shown that different species belonging to the Magnoliids — a basal group within the flowering plants — and the rest, which belongs to much more recent lineages, are endowed with molecules that can control tropical parasites. This is evident that the molecules of interest for controlling these diseases are widely distributed in the lineages of flowering plants.

## Authors’ Contributions

WC, FA, and SR conceived the study designed. FA collected plant material. CH, JC, and IDV performed the experiment. AY and IVT analyzed the data. WC and SR drafted and revised the manuscript. All authors read and approved the final manuscript.
